# Molecular genetics of naringenin biosynthesis, a typical plant secondary metabolite produced by *Streptomyces clavuligerus*

**DOI:** 10.1186/s12934-015-0373-7

**Published:** 2015-11-09

**Authors:** Rubén Álvarez-Álvarez, Alma Botas, Silvia M. Albillos, Angel Rumbero, Juan F. Martín, Paloma Liras

**Affiliations:** Microbiology Section, Department of Molecular Biology, Faculty of Biology and Environmental Sciences, Vegazana Campus, University of León, León, 24071 Spain; Institute of Biotechnology, INBIOTEC, Av. Real 1, León, 24006 Spain; Organic Chemistry Department, University Autónoma of Madrid, Cantoblanco, 28049 Madrid, Spain

**Keywords:** Naringenin, Secondary metabolism, *Streptomyces clavuligerus*, Histidine/phenylalanine ammonium lyase

## Abstract

**Background:**

Some types of flavonoid intermediates seemed to be restricted to plants. Naringenin is a typical plant metabolite, that has never been reported to be produced in prokariotes. Naringenin is formed by the action of a chalcone synthase using as starter 4-coumaroyl-CoA, which in dicotyledonous plants derives from phenylalanine by the action of a phenylalanine ammonia lyase.

**Results:**

A compound produced by *Streptomyces clavuligerus* has been identified by LC–MS and NMR as naringenin and coelutes in HPLC with a naringenin standard. Genome mining of *S. clavuligerus* revealed the presence of a gene for a chalcone synthase (*ncs*), side by side to a gene encoding a P450 cytochrome (*ncyP*) and separated from a gene encoding a Pal/Tal ammonia lyase (*tal*). Deletion of any of these genes results in naringenin non producer mutants. Complementation with the deleted gene restores naringenin production in the transformants. Furthermore, naringenin production increases in cultures supplemented with phenylalanine or tyrosine.

**Conclusion:**

This is the first time that naringenin is reported to be produced naturally in a prokariote. Interestingly three non-clustered genes are involved in naringenin production, which is unusual for secondary metabolites. A tentative pathway for naringenin biosynthesis has been proposed.

**Electronic supplementary material:**

The online version of this article (doi:10.1186/s12934-015-0373-7) contains supplementary material, which is available to authorized users.

## Background

*S. clavuligerus* ATCC 27064 is an industrially relevant actinobacteria, used for the production of clavulanic acid (CA) [1]. This strain has a peculiar carbohydrate metabolism [2] and is able to produce in addition to CA other bioactive compounds such as cephamycin C [3], clavams [4] and holomycin [5, 6]. Interestingly, sequencing of the *S. clavuligerus* genome revealed that this organism has potential ability to produce a wealth of additional secondary metabolites [7]. Up to 25 clusters for putative secondary metabolites are located in the 6.8 Mb chromosome of *S. clavuligerus* and 23 more are present in the 1.8 Mb megaplasmid pSCL4 [7, 8]. They include ten clusters containing genes for nonribosomal peptide synthetases (NRPS), six clusters with genes for polyketide synthases (PKS), as well as NRPS-PKS hybrid clusters and genes for putative terpene synthesizing enzymes.

Gene clusters encoding novel bioactive products are of utmost interest. The homology of some clusters with previously studied orthologous in other *Streptomyces* species suggest that *S. clavuligerus* might produce staurosporine, moenomycin, indigoidine, and enediyne-like compounds, some of which are antitumor agents. Since most of these compounds have never been detected in *S. clavuligerus* cultures they must be silent clusters that are only expressed in response to specific nutritional or physiological signals, or in response to elicitors [9, 10].

Chalcone synthases are type III polyketide synthases involved in the condensation of different CoA-activated precursor starter units (i.e. phenylpropenoids) to produce flavonoid-type chalcones. They are very frequent in fungi and in plants, where they serve as precursors of different types of flavonoids [11] but are rare in actinomycetes. Some types of flavonoid intermediates, e.g. naringenin, appeared to be restricted to plants. An interesting question is whether there are chemical structures of secondary metabolites restricted to plants or if many of them can be found in some actinobacteria. In this work we describe that *S. clavuligerus* is able to produce naringenin, a flavanone important as antioxidant [12], described to act as antiinflamatory, chemoprotective and antitumor agent [13, 14].

**Fig. 1:**
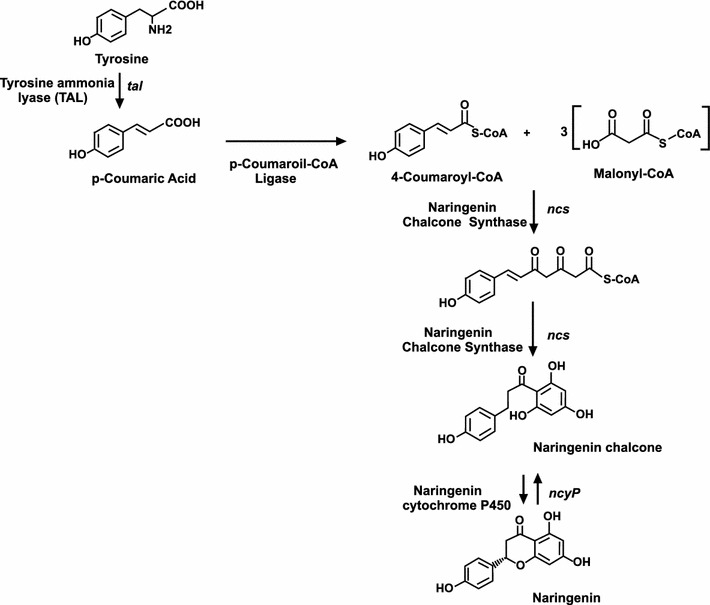
Proposed naringenin biosynthesis pathway in *S. clavuligerus*. The names of the intermediate compounds, enzymes involved and genes are indicated. Naringenin biosynthesis starter in plants is usually phenylalanine and there are additional steps for the hydroxylation of cinnamic acid to produce *p*-coumaroic acid

Naringenin is naturally produced in several plants, especially in grapefruit, and its biosynthesis has been studied in *Medicago*, parsley and other plants (Fig. 1) but it is unknown if the same pathway occurs in *S. clavuligerus*. The starter unit for naringenin biosynthesis is 4-coumaroyl-CoA, which in dicotyledonous plants derives from phenylalanine by the action of a phenylalanine ammonia lyase (PAL). This compound is hydroxylated at carbon 4 by a cinnamate-4-hydroxylase and activated by a CoA-dependent ligase [15]. Monocotyledonous plants might use, in addition, tyrosine as substrate producing directly *p*-coumaric acid without the need of the cinnamate-4-hydroxylase. A related enzyme, encoded by the *encP* gene has been detected in *Streptomyces maritimus* and found to convert phenylalanine in cinnamic acid, required for enterocin biosynthesis [16], which lacks the hydroxyl group at the C-4 position, but production of naringenin in prokaryotes has never been reported. It was therefore of interest to study if the naringenin pathway and putative gene cluster(s) in *S. clavuligerus* is similar to that of plants and whether it is expressed under some specific conditions.

## Results

### Characterization of naringenin as a novel product of *S. clavuligerus* ATCC 27064

**Fig. 2:**
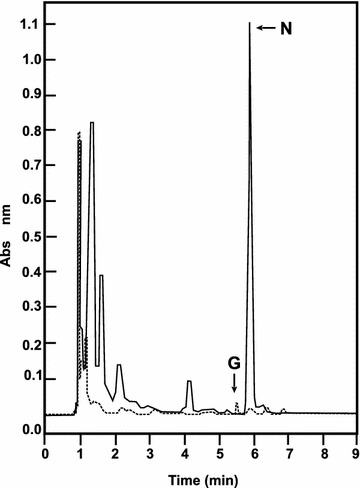
HPLC analysis of culture broths of *S. clavuligerus*. The broth of cultures grown in SA medium (*discontinuous lane*) and TSB medium (*continuous line*) were analyzed as indicated in “Methods”. The peak with a retention time of 6.0 min coincides with pure commercial naringenin (N). Pure genistein (G) used as standard has a retention time of 5.4 min

In the course of a comparative HPLC study of the compounds produced by *S. clavuligerus* in TSB and SA media a large absorbance peak was found in cultures grown in TSB medium that was not present in the broth of SA grown cultures. TSB is a tryptic hydrolysate of soybean meal rich in plant-derived amino acids whereas SA contains only asparagine as nitrogen source. The peak eluted at a retention time of 6 min under the HPLC conditions used in the assay (Fig. 2) and corresponds to an extracellular compound that is also present in extracts of washed cells at about 1 % of the concentration found in the broth. No appreciable amount of the compound was found in the unseeded TSB medium indicating that it is formed *de novo* in *S. clavuligerus* cultures.

To purify the compound batch cultures were carried out for 72 h to obtain a total of 2.4 l of culture broth. The centrifuged culture supernatant was concentrated under vacuum and extracted with ethyl acetate as indicated in “Methods”. After repeated HPLC injections the compound eluting at 6.0 min was collected and lyophilized to obtain 5 mg of pure material that was used for the chemical characterization of the molecule.

### The compound produced by *S. clavuligerus* as identified by LC–MS and NMR is naringenin and coelutes in HPLC with authentic naringenin

**Fig. 3:**
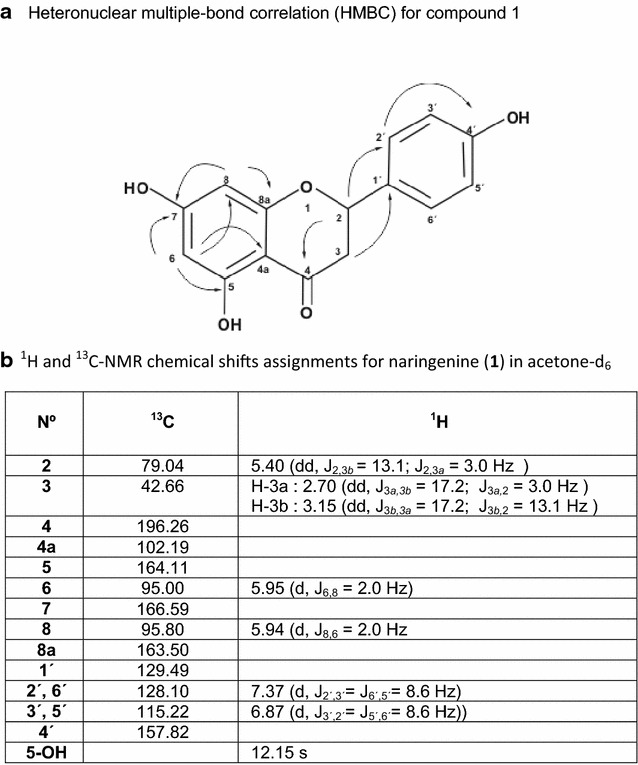
NMR analysis for the purified compound. **a** Heteronuclear multiple-bond correlation. **b** ^1^H and ^13^C-NMR chemical shifts assignments for naringenin in acetone-d6

A mass [M+H]^+^ of 273 and a molecular formula of C_15_H_12_O_5_ was obtained from LC–MS and from the ^13^C-NMR analysis. Examination of NMR data analysis (Fig. 3) suggested that compound 1 was a flavanone. Its ^1^H-NMR spectrum revealed characteristic resonances of aromatic protons including four proton signals of the B-ring appearing as two doublets at δ 7.37 and 6.87 ppm with J = 8.6 Hz due to *ortho*- coupling that were assigned to H-2′, 6′ and H-3′, 5, respectively; the two *meta*-coupled protons at δ 5.95 and 5.94 ppm with J = 2.0 Hz were assigned to H-6 and H-8. The presence of the quelated signal of 5-OH with the 4-keto group at δ 12.15 and signals at δ 5.40 (1H, dd, J = 13.1 and 3.0 Hz), δ 2.70 (1H, dd, J = 17.2 and 3.0 Hz) and δ 3.15 (1H, dd, J = 17.2 and 13.1 Hz), in conjunction with the ^13^C-NMR signals at δ 79.04 and 42.66 ppm, inferred from HMQC spectrum, pointed to the presence of an –O–CH–CH_2_–CO– system in the C-ring of the flavanone component. This conclusion was reinforced by the correlation of peak signals at δ 3.15 and 2.70 ppm with the signal at δ 5.40 observed in the ^1^H-^1^H COSY spectrum.

The assignment of the signals of the ^13^C-NMR spectrum, corresponding at δ 95.00 (C-6), δ 95.8 (C-8), δ 115.22 (C-3′ and C-5′) and δ 128.10 (C-2′ and C-6′) was carried out by using the HSQC-HMQC spectrum, which showed a correlation peak via ^1^J_C-H_. Other heteronuclear correlations deduced from the HMBC spectrum (Fig. 3) via ^2^J_C-H_ and ^3^J_C-H_, were used to assign the signals corresponding at δ 196.26 (C-4), δ 157.82 (C-4′), δ 166.59 (C-7), δ 164.11 (C-5), δ 163.50 (C-8a), δ 129.49 (C-1′) and δ 102.19 (C-4a).

All these NMR data together with the mass spectrum suggested the structure of naringenin [17] for compound 1. The produced compound was different from genistein, a plant flavonoid intermediate similar to naringenin but carrying an additional double bond; both compounds were clearly resolved by HPLC (retention times of 5.4 and 6.0 min for genistein and naringenin, respectively).

### Production of naringenin is stimulated by aromatic amino acids

**Fig. 4:**
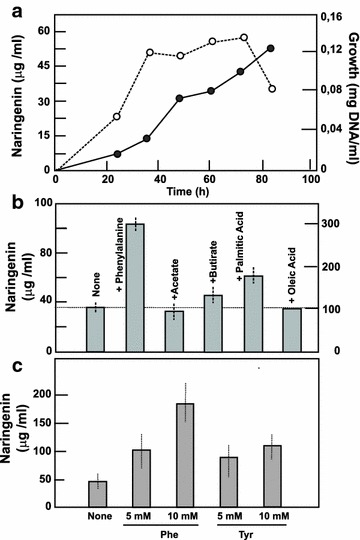
Production of naringenin by *S. clavuligerus*. **a** Profile of a culture of *S. clavuligerus* grown in TSB medium. Growth (*open circles*), naringenin production (*closed circles*). **b** Naringenin production at 84 h of cultures by *S. clavuligerus* ATCC 27064 in the absence and presence of phenylalanine, acetate, butyrate, palmitic acid or oleic acid at 5 mM concentration. **c** Production of naringenin in TSB cultures supplemented with phenylalanine or tyrosine at 5 and 10 mM concentration

Production of naringenin in TSB medium increases in parallel to the growth of the culture reaching the maximum level at 84 h (Fig. 4). TSB is a complex medium; however, it might not have the adequate precursor requirements for naringenin production. Since this compound is known to be formed from malonate and tyrosine or phenylalanine in different plants, we supplemented the TSB medium separately with different organic acids (acetic, butyric, palmitic and oleic acid, as sodium salts) at 5 mM concentration and the final production of naringenin was measured in each case. Acetate and oleic acid did not improve naringenin production, but butyrate and palmitic acid improved it 1.15- and 1.63-fold, respectively.

In addition, cultures were supplemented with phenylalanine or tyrosine at 5 and 10 mM concentration (Fig. 4c). Tyrosine improved the naringenin production by 1.83- and 2.32-fold at 5 and 10 mM concentration, respectively. The best production was observed by addition of phenylalanine, resulting in a naringenin increase of 2.16- and 3.7-fold respectively, at 5 and 10 mM concentration (up to 184 µg/ml). This concentration is much higher than the production obtained by a multistep engineered *E. coli* strain [14] indicating that *S. clavuligerus* ATCC 27064 is a good naringenin producer.

### Bioinformatic analysis of genes putatively encoding naringenin biosynthetic enzymes in the *S. clavuligerus* genome

The putative pathway for naringenin biosynthesis (Fig. 1) requires the action of a naringenin chalcone synthase (Ncs) condensing the *p*-coumaroyl-CoA starting unit and three malonyl-CoA molecules. Therefore, using the sequence of *Glycine max* naringenin chalcone synthase (CAA38456) we searched *S. clavuligerus* genome for homologous genes using the BLAST Program. A single match corresponded to *S. clavuligerus* SCLAV_5492; this gene (named *ncs*) encodes a 351 amino acid protein with characteristics of type III polyketide synthases and a 29 % identity (44 % functionally conserved amino acids) along the entire sequence with *G. max* naringenin chalcone synthase, hereafter named Ncs. Interestingly, this chalcone synthase is closely related to type III PKSs of the RppA family occurring in many *Streptomyces* species (see “Discussion”).

Inmediately upstream of *ncs* we found a gene, SCLAV_5491, encoding a 454 amino acid cytochrome P_450_ oxygenase that will be named hereafter *ncyP*. Genes encoding P_450_ oxygenases are frequently present in secondary metabolites gene clusters and we postulated that *ncyP* might encode the enzyme required for the chalcone cyclization (see deletion and complementation experiments). This cytochrome P_450_ gene is more closely related to *rppA*-linked P_450_'s in several *Streptomyces* species than to other P_450_'s genes present in *S. clavuligerus* genome (see “Discussion”).

Genes encoding proteins analogous to Ncs and NcyP are present with the same organization in *S. coelicolor* (SCO1206 and 1207), *S. lividans* (Sli_1485 and 1486) or *S. griseus* (SGR_6619 and 6620) while in *S. venezuelae* (SVEN_5366 and 5367) are located tail to tail, and in *S. avermetilis* (SAV_1171 and SAV_7131) are separated in the genome. The linkage of the *ncs* and *ncyP* genes may be relevant for efficient formation of naringenin (see “Discussion”). A tyrosine, at position 224, conserved in the *S. clavuligerus* Ncs protein, might be involved in the recognition of the first (starter) unit of p-coumaroil-CoA, or malonyl-CoA, as proposed in the RppA synthase of *S. coelicolor* [18, 19].

### Location of genes putatively involved in the formation of the 4-coumaroyl-CoA precursor

Naringenin is formed from a 4-coumaric acid starting unit. In order to find genes encoding the enzyme required for 4-coumaroyl biosynthesis, the *encP* gene of *Streptomyces maritimus,* encoding a phenylalanine/tyrosine ammonia lyase [16], was used to search *S. clavuligerus* genome. One single matching gene, SCLAV_5457, was found. This gene, located 47.3 kb upstream of the *ncs*-*ncyP* cluster encodes a 559 amino acid protein with 37 % amino acids identity (51 % conserved residues) to EncP along the whole sequence. This protein is related to TAL or PAL phenylalanine/tyrosine ammonia lyases and the gene will be named *tal* hereafter [20]. These enzymes are able to deamine phenylalanine to form cinnamic acid and tyrosine to produce coumaric acid. A short chain dehydrogenase/reductase encoded by SCLAV_5458, located downstream of the former gene may act on the double bond of the propenoyl side chain of 4-coumaric acid to reduce the double bond.

Activation of coumaric acid is likely to be mediated by the action of the aryl-CoA ligase encoded by SCLAV_3408. This last enzyme contains an ATP binding site (TGDIL, amino acids 386–390) for activation of the aromatic acid as an acyl-adenylate and is similar to coumaroyl-CoA ligases of plants and *Penicillium chrysogenum* [20–22] and to the benzoyl-CoA ligase of *S. maritimus* involved in the biosynthesis of enterocin [16].

### Deletion of *S. clavuligerus ncs* and *ncyP* genes blocks naringenin biosynthesis

**Fig. 5:**
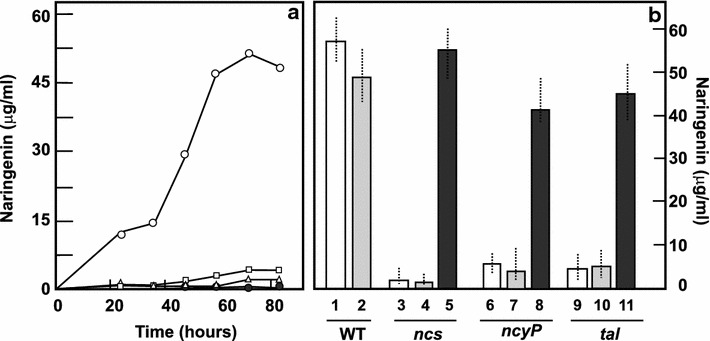
Production of naringenin by *S. clavuligerus* deleted mutants and complemented strains. **a** Production of naringenin by *S. clavuligerus* 27064 (*open circles*), *S. clavuligerus ncs::aac* (*closed circles*), *S. clavuligerus ncyP::aac* (*open triangles*), *S. clavuligerus tal::aac* (*open squares*). **b** Production of naringenin by *S. clavuligerus* deleted and complemented strains at 96 h of growth. (1) *S. clavuligerus* ATCC27064, (2) *S. clavuligerus* [pAV11], (3) *S. clavuligerus* Δ*ncs::aac* (4) *S. clavuligerus* Δ*ncs::aac* [pAV11], (5) *S. clavuligerus* Δ*ncs::aac* [pAV11-*ncs*], (6) *S. clavuligerus* Δ*ncyP::aac* (7) *S. clavuligerus* Δ*ncyP::aac* [pAV11], (8) *S. clavuligerus* Δ*ncyP::aac* [pAV11-*ncyP*], 9) *S. clavuligerus* Δ*tal::aac,* (10) *S. clavuligerus* Δ*tal::aac* [pAV11], and (11) *S. clavuligerus* Δ*tal::aac* [pAV11-*tal*]

In order to elucidate if the *ncs* or *ncyP* genes are involved in naringenin biosynthesis we deleted, separately, each of these genes using the Redirect procedure. Seven clones of *S. clavuligerus* ∆*ncs::aac* were obtained. The deleted strains showed the same pigmentation, aerial mycelium and spores formation as the parental strain. However, when tested for naringenin biosynthesis in TSB liquid cultures, production of naringenin was drastically reduced to about 9 % of that in the parental strain, in the seven clones at 84 h cultures (Fig. 5).

Deletion of *ncyP* produced two exconjugants designated as *S. clavuligerus* ∆*ncyP::aac*. Both mutants produced only traces of naringenin, about 4 % of the level of the parental strain indicating that *ncyP* (but not other P_450_ oxygenases present in the cell) is strictly required for naringenin biosynthesis. Therefore, for the first time it is demonstrated that both genes, *ncs* and *ncyP*, are involved in naringenin formation.

### Complementation of the *ncs* and *ncyP* genes in the deleted mutants restores naringenin production

*Streptomyces clavuligerus* mutants ∆*ncs::aac* and ∆*ncyP::aac* were complemented with each of the deleted genes subcloned in the integrative plasmid pAV11 downstream of the anhydrotetracycline-inducible *tcp80* promoter. The complemented strains were grown in TSB medium in the presence of anhydrotetracycline (10 µg/ml) to induce the *tcp80* promoter [23]. Production of naringenin was measured after 96 h of culture in all strains. *S. clavuligerus* ATCC 27064 produced 58 µg/ml. The control transformant with the integrated empty plasmid, *S. clavuligerus* [pAV11], produced a slightly lower naringenin level (48 µg/ml). The production in the deleted mutants and their controls transformed with the empty plasmid dropped to 2–7 µg/ml in the different strains. However, complementation of the *ncs*-deleted mutant resulted in restoration of naringenin production (to 55 µg/ml) and complementation of the *ncyP*-deleted mutant resulted in naringenin concentrations of 42 µg/ml (Fig. 5b). These results confirmed the involvement of *ncs* and *ncyP* in naringenin biosynthesis.

### *S. clavuligerus* Δ*tal::aac*, deleted in the *tal* gene, does not produce naringenin but production is restored by complementation

The *tal* gene, encoding a TAL/PAL enzyme was cloned from *S. clavuligerus* genomic DNA. Using oligonucleotides tal_F and tal_R a 1929 bp DNA fragment was amplified, sequenced and used to locate a cosmid containing the *tal* gene. Two cosmids D6-6 and D9-10 gave positive hibridization with the 1.9 kb DNA fragment. The sequencing of the insert of both cosmids showed that the *tal* gene was in a central position in cosmid D9-10 which was used for further studies. Seven apramycin resistant kanamycin sensitive clones, deleted in *tal*, were obtained by the Redirect technique. The seven clones were identical by restriction mapping, PCR amplification and sequencing confirming that the *tal* gene was substituted by the *aac* gene; the deleted strain was named *S. clavuligerus* Δ*tal::aac*. The *tal* mutants showed a normal behavior in sporulation and aerial mycelium formation in TSB and ME media. When they were grown in TSB liquid cultures the naringenin production at 96 h was lower than 6 % in comparison to the wild type strain. Complementation of one of these mutants with the *tal* gene results in a transformant producing 46 µg/ml, which is 95 % of the production of the control strain *S. clavuligerus* [pAV11] (Fig. 5b). This confirms that the ammonia lyase encoded by *tal* is involved in naringenin formation, probably by using tyrosine or phenylalanine as substrate(s) to form the 4-coumaric acid precursor unit. In this respect, *S. clavuligerus* is similar to monocotyledonous plants that are able to use a TAL to form directly *p*-coumaric acid and differs from dicotyledonous plant cells which form coumaric acid through a hydroxylation of trans-cinnamic acid by the enzyme cinnamate-4-hydroxylase. Our study showed that *S. clavuligerus* genome does not contain a cinnamate-4-hydroxylase.

### RT-PCR transcription analysis of the genes involved in naringenin biosynthesis

**Fig. 6:**
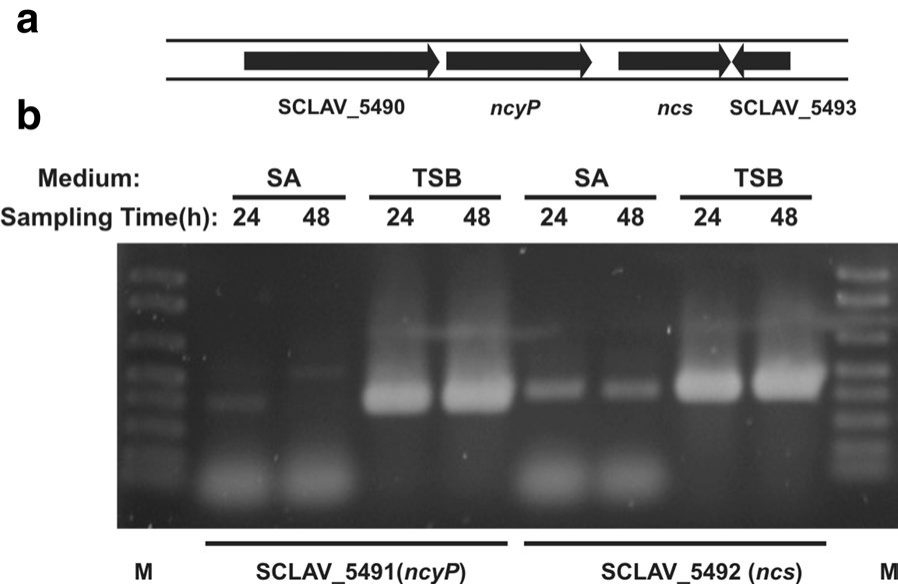
Organization and expression of the *ncs* and *ncyP* cluster of *S. clavuligerus.* **a** Organization of the *ncs*-*ncyP* gene cluster. **b** Expression of the *ncyP* and *ncs* genes in SA and TSB medium at 24 and 48 h of growth as detected by RT-PCR after 35 cycles of amplification in the conditions indicated in “Methods”

Expression of *ncs* and *ncyP* in two different media (SA and TSB) was tested by RT-PCR at 24 and 48 h of culture. As shown in Fig. 6 a very weak amplification band of these two genes is observed in cultures grown in SA medium while a strong amplification signal was found in TSB medium what corroborates our initial results on production of naringenin in each of these media. Expression of these genes might be induced by soja-derived compounds in the triptic soja broth as reported for other P_450_ genes [24]. Using the oligonucleotides indicated in the Additional files 1, 2 an RT-PCR analysis was done for the intergenic *ncs*-*ncyP* region. No amplification of the intergenic fragment was detectable (not shown) in RNA samples in which both *ncs* and *ncyP* were clearly amplified, indicating that each of these genes was expressed as a monocistronic unit.

To determine whether the stimulatory effect observed by phenylalanine and tyrosine addition to the cultures was a precursor availability effect, or if it was due to an induction effect, quantification by qRT-PCR of the *ncs*, *ncyP* and *tal* transcripts in control cultures and cultures supplemented with phenylalanine or tyrosine (each at 5 and 10 mM concentration) was made. No significant differences in the transcription of the three genes was observed following phenylalanine or tyrosine addition, and only a very weak stimulation of the *ncs* transcription was observed in the presence of phenylalanine. Therefore the stimulatory effect of these two aromatic amino acids on naringenin production is likely due to a precursor effect and not to induction of the naringenin biosynthetic genes.

### RppA versus Ncs: *Streptomyces coelicolor* forms tetrahydroxynaphthalene but does not produce naringenin

Bacterial type III polyketide synthases are a group of PKSs that contain two KS domains and catalyze repeated condensations of malonyl/CoA units. They are similar to plant chalcone synthases [25] in amino acid sequence, although the plant chalcone synthases are usually larger enzymes. Some type III PKSs have been described in soil dwelling *Streptomyces* species and some related actinobacteria [26]. Several of these PKSs are involved in the production of red-brown pigments and consequently the encoding gene was named *rppA* [27–29]. The best known of these PKSs are the 1,3,6,8 tetrahydroxynaphtalene (THN) synthases of *S. griseus* and *S. coelicolor* encoded by *rppA* genes. In the formation of THN the RppA PKS uses malonyl-CoA as starter unit and three additional malonyl-CoA as elongation units [30]. In some *Streptomyces* species the THN is further oxidized to flaviolin [26]. Although *S. clavuligerus* Ncs and *S. coelicolor* RppA are related enzymes our study showed that *S. clavuligerus* does not produce red brown THN pigment and, additionally, the Δ*ncs* mutant culture broth remains of the same colour as the wild type cultures. Moreover, *S. coelicolor* A3(2), that is well known to synthesize THN does not produce naringenin when cultured in TSB medium under conditions in which *S. clavuligerus* produces this compound.

It was therefore of interest to know if *S. coelicolor* is able to produce naringenin when supplied, exogenously or endogenously, with *p*-coumaric acid. To test if the *S. coelicolor* RppA might be able to produce naringenin, two different experimental approaches were followed. First, *S. coelicolor* M145 cultures were supplemented with coumaric acid (5 mM). Second, *S. coelicolor* M145 was transformed with the *S. clavuligerustal* gene (SCLAV_5457) expressed from the anhydrotetracyclin inducible *tcp830* promoter in plasmid pAV11 to provide endogenous coumaric acid. Cultures of *S. coelicolor* [pAV11-*tal*] were supplemented with phenylalanine, tyrosine or coumaric acid (5 mM) and compared with cultures of *S. coelicolor* [pAV11] transformed with the empty plasmid, grown in the same conditions. None of this strategies resulted in detectable levels of naringenin production by *S. coelicolor* suggesting that the RppA chalcone synthase is unable to use in vivo efficiently *p*-coumaroyl-CoA instead of malonyl-CoA as starter unit.

## Discussion

In plants and in *S. clavuligerus*, as shown in this article, the initial product of the Ncs is the naringenin chalcone, that plants usually converted later to flavonoids. Naringenin is synthesized by cyclization of a tetraketide formed by condensation of a *p*-coumaroyl-CoA starter and three malonyl-CoA elongation units (Fig. 1). The cyclization mechanism catalyzed by Ncs that leads to the formation of the pyrone ring of naringenin (Fig. 1) is different from the aromatization mechanism that results in the formation of THN, which involves a C–C condensation with the free carboxyl group of the starter malonyl-CoA.

The *ncs*-*ncyP* gene cluster and the *tal* gene, although located in two separate positions (47.3 kb apart) in the genome cooperate in the formation of the *p*-coumaric acid precursor unit and in the assembly of the naringenin molecule, an usual feature of genes for secondary metabolites that normally are clustered in *Streptomyces.*

The crystal structure of some CHSs [31, 32] revealed an important role of the size of the cavity of the active center in the selection of the starter unit, and therefore, of the product of the pathway. This may explain the different products of NCS versus other related type III PKSs.

*Streptomyces* RppA’s were hypothesized to have a small active center cavity that allowed only the entry of small acyl-CoA derivatives such as malonyl-CoA [33] different from plant chalcone synthases that use bulky starter units such as *p*-coumaroyl-, caffeyl- or *trans*-cinnamoyl-CoA.

The amino acid sequence of *S. clavuligerus* Ncs and the RppAs of *S. avermitilis* and *S. coelicolor* are very similar (76 and 74 % identity, respectively). All these proteins posseses the four amino acid residues characteristics of chalcone synthases active center (Cys^138^, Phe^188^, His^270^ and Asn^303^ in Ncs enzyme) [31]. The sequence of amino acids around the active center is different in *Streptomyces* and plant chalcone synthetases (Additional file 1: Figure S1), but 50 % of these amino acids are conserved. Therefore, *S. clavuligerus* Ncs is a member of the RppA family and is phylogenetically closer to *Streptomyces* than to plant chalcone synthases in spite of giving a final plant typical product. However, *S. clavuligerus* does not produce significant amounts of THN or THN-derived pigments indicating that *p*-coumaroyl-derived naringenin is the major product of this enzyme. Furthermore, the *S. clavuligerus* Δ*ncs* mutant is not albino at difference of the *S. griseus* Δ*rppA* mutant and does not change in colour with respect to its parental strain.

The Tyr^224^ in the entry channel of RppA’s was proposed as the amino acid determining the selective use of malonyl-CoA as starter unit, although this hypothesis was later disputed by new evidence provided by a set of mutants in the Tyr^224^ amino acid [18]. Interestingly, the *S. clavuligerus* Ncs has a conserved Tyr at position 224 thus indicating that this amino acid is not strictly required for the entry of *p*-coumaroyl-CoA as starter unit of the polyketide, which is in agreement with the finding of Li et al. [18].

Linked to *ncs* in *S. clavuligerus* genome is a *ncyP* gene encoding a P_450_ oxygenase that is strictly required for naringenin biosynthesis. Similarly the P_450_ gene associated with *rppA* in *S. griseus* (known as *rppB*) is also required for THN formation [32, 34]. Our studies show that those two genes separatedly transcribed whereas in *S. griseus* they appear to be co-transcribed and translationally coupled, suggesting that both proteins are likely to form a functional complex. Cortés et al. [27] suggested that the RppA associated P_450_ has a role in cross-linking of THN molecules and this hypothesis was later supported by the study of strains overexpressing *rppB* that form dimers and trimmers of flavolin, the quinone derived from THN. Moreover, this P_450_ oxygenase has probably a role as a helper in the first cyclization of the tetraketides, both in THN and naringenin biosynthesis, what explains the essential role of these P_450_ oxygenases in the formation of THN and naringenin, that later proceed by different final cyclization processes.

### Tyrosine ammonia lyase and formation of *p*-coumaroyl-CoA

Phenylalanine and tyrosine ammonia lyases belong to a family of aromatic amino acid ammonia lyases that are closely related to bacterial histidine ammonia lyases and aminomutases [35]. These enzymes catalyze the non-oxidative deamination of aromatic amino acids resulting in the formation of trans-*p*-coumaric acid when the substrate is l-tyrosine or trans-cinnamic acid when the substrate is phenylalanine. The biosynthesis of trans-cinnamic acid in dicotyledonous plants is catalyzed by a large PAL that works as a tetramer. A separate enzyme cinnamate 4-hydroxylase introduces the hydroxyl group at position 4 of the aromatic ring. In monocotyledonous plants both reactions are catalyzed by a single enzyme with TAL/PAL enzyme that uses both tyrosine and phenylalanine as substrates [36]. The bacterial aryl ammonia lyases are smaller (about 400 amino acids) tetrameric ammonia lyases [35]. Based on the crystal structure of the *Rhodobacter* histidine ammonia lyase and on the kinetics data of some mutant enzymes Louie and coworkers [36] proposed that His^89^ has an important role in this enzyme recognition of histidine or aromatic amino acids as substrates, since His^89^ forms hydrogen bridges with the substrates of the enzyme.

**Fig. 7:**
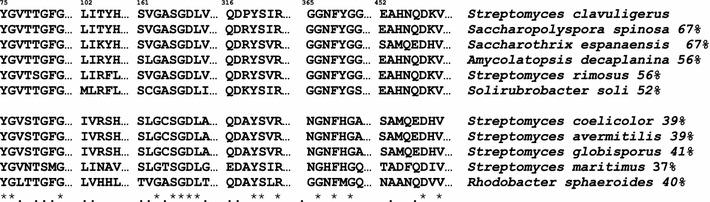
Partial amino acid sequence alignment of regions known to form the active site of TAL proteins. Above are indicated the amino acid positions in *S. clavuligerus* SCLAV_5457 sequence. At the right side is shown the % of amino acids identity of the whole protein. The sequences used in this figure are ammonia lyases from *Saccharopolyspora spinosa* (WP_029535616.1), *Saccharothris espanaensis* (WP_015102494), *Amycolatopsis decaplanina* (WP_007032994), *Streptomyces globisporus* (WP_029182093.1), *Streptomyces rimosus* subsp. *rimosus* ATCC 10970 (ELQ83456.1), *Streptomyces maritimus* EncP (gb|AAF81735.1), *Solirubrobacter soli* (WP_028063475.1), *Streptomyces coelicolor* (WP_629085.1), *Streptomyces avermitilis* (WP_010984755.1) and *Rhodobacter sphaeroides* (WP_011339422.1)

*S. clavuligerus* TAL, as the homologous enzyme of *Saccharothrix spanaensis* involved in the biosynthesis of saccharomicin [37], has a rather different amino terminal region when compared with the *Rhodobacter* enzyme, although six motifs described in the *Rhodobacter* enzyme to interact with the substrate are conserved to some degree (Fig. 7). The H^89^ present in one of these motifs in the *Rhodobacter* ammonia lyase corresponds to Y^105^ in *S. clavuligerus* TAL; this Y^105^ is conserved (or substituted by Phe) in a small group of actinobacteria which have ammonia lyases with 50–60 % identity to *S. clavuligerus* TAL, while the bulk of *Streptomyces* ammonia lyases have about 40 % identity with the *S. clavuligerus* enzyme, and differ in that they have an IVRSH sequence with serine at the corresponding 105 position. Using in vitro reactions with the *Saccharothrix* enzyme, that has a LIKYH domain carrying tyrosine [37] it has been clearly demonstrated that the product of this ammonia lyase is *p*-coumaric acid and not trans-cinnamic acid and the substrate used is l-tyrosine. Similar observations have been made for tyrosine amino mutase of *Streptomyces globisporus* that forms *p*-coumaric acid as an intermediate in the α- to β- tyrosine isomerization and has 37 % identity to the *S. clavuligerus* TAL.

Aromatic amino acid ammonia lyases were thought to contain a dehydroalanine prosthetic group derived from serine (in the *Rhodobacter sphaeroides* HAL protein) but later molecular genetics and spectrophotometric studies [38, 39] established that the prosthetic group is methylidene-imidazol-5-one (MIO). This molecule is formed by autocatalytic dehydration and cyclization of the internal tripeptide ala-ser-gly that in *S. clavuligerus* TAL corresponds to amino acids A^164^-S^165^-G^166^ (Fig. 7). This tripeptide precursor of the prosthetic MIO group is present in all putative aromatic amino acid ammonia lyases and amino acid mutases in several *Streptomyces* species. The prosthetic group MIO of aromatic amino acid ammonia lyases is also present in the *S. clavuligerus* TAL protein as deduced from spectrophotometric studies [Álvarez-Álvarez, unpublished results).

In summary, three enzymes Ncs, NcyP and Tal are involved in naringenin formation in *S. clavuligerus*, a strain that does not produce the THN pigment in spite of the similarity of Ncs to the *S. coelicolor* RppA protein.

## Methods

### Strains and culture conditions

The wild type strain, *Streptomyces clavuligerus* ATCC 27064 was used to purify naringenin. Strains *S. clavuligerus* ∆*ncs::aac, S. clavuligerus* ∆*ncyP::aac* and *S. clavuligerus* Δ*tal::aac* were obtained from the wild type strain by the REDIRECT Technique. *Streptomyces coelicolor* [pAV11-*tal*] and *S. coelicolor* [pAV11] derive from *S. coelicolor* M145 and were used to study possible naringenin production.

All the strains were grown at 28 °C in an orbital shaker incubator at 220 rpm for 24 h in 500 ml baffled flasks containing 100 ml of TSB medium. A 5 % (v/v) of this seed culture was used to inoculate TSB (trypticaseine broth) or SA (starch-asparragine) cultures grown in the same conditions [40]. All the cultures were made in triplicate flasks. The TSB was supplemented with tyrosine, phenylalanine or with organic acids (acetate, butirate, oleic acid, palmitic acid) as sodium salts, when required. Cultures of transformants strains were supplemented with kanamycin (50 µg/ml), anhydrotetracycline (10 µg/ml), hygromycin (50 µg/ml), apramycin (50 µg/ml) or nalidixic acid (25 µg/ml) when required. Complex ME medium [41] was used for sporulation purposes.

### Naringenin purification by HPLC

Naringenin was purified by HPLC using an Alliance 2695 HPLC with a refrigerated autosampler, a column furnace and a photodiode array detector (PDA) 2998 (Waters Corp.). Naringenin was quantified at its maximum absortion peak (290 nm). The system was equipped with a 3.5 μm particle size XBridge™ C18 (2.1 × 150 μm, Waters Corp.) column.

The culture samples were centrifuged at 13,000 rpm for 5 min and the supernatants filtered through sterile 0.2 μm polyethersulphone (PES) syringe filters (VWR, Intl.). The whole sample (100 ml) was loaded in multiple injections. HPLC conditions were as follows: column temperature 40 °C; the flow was kept at 0.32 ml/min from 0 to 2 min and then increased gradually to reach 0.40 ml/min at 10 min. A mixture of Milli Q water with 0.01 % trifluoroacetic acid (A) and acetonitrile (B) were used as solvent with the following program: 0–2 min increase from 20 to 30 % B and kept at 30 % B thereafter. The peak eluting at 6 min from repeated injections was collected and pooled and the solvent from the pooled preparation was evaporated under low pressure (SpeedVac Savant Sc110, Thermo Sci.). The concentrated solution was lyophilized and kept at −20 °C until NMR analysis and identification. Pure naringenin and genistein (Sigma Ch. Co.) were used as standards.

### NMR analysis

NMR spectra were recorded in metanol-d_4_ at room temperature using a Bruker WM 500 spectrometer [500 MHz (^1^H NMR) and 125 MHz (^13^C NMR)]. Chemical shifts are given on the δ-scale and were referenced to the solvent (δc = 49.0 ppm) and to the TMS as internal standard. The pulse programs of the following 2D experiments were taken from the Bruker software library and the parameters were as follows: 500/125 MHz gradient-selected HMQC spectra [1]: relaxation delay D_1_ = 1.5 s; 500/125 MHz gradient-selected HMBC spectra [1]: relaxation delay D_1_ = 1.5 s; evolution delay D_2_ = 3.33 ms; delay for evolution of long range coupling D_6_ = 60 ms. 500 MHz gradient-selected ^1^H, ^1^H COSY spectra [2]: relaxation delay D_1_ = 1.5 s; 90° pulse for ^1^H.

### Mass spectrometry

The mass spectra were determined by HPLC–MS (Agilent Technologies). Using a HP-1100 Series HPLC and MS: 6120 Quadrupole LC/MS.

### Extraction and purification of RNA

RNA was extracted from 1.5 ml samples taken at 24 and 48 h of growth in TSB and SA media. Two volumes of RNA-protect (Qiagen) were added to the samples. The commercial system Qiagen, RNAeasy was used following the manufacturer instructions as described previously [40].

### RT-PCR

For RT-PCR amplification the final 20 µl reaction contained RNA (200-300 ng), reaction mixture 1×, 0.5 mM each oligonucleotide (see Additional file 2: Table S1); 5 % DMSO; *SuperScriptTM II* Reverse Transcriptase and *Platinum*^*®*^*Taq* (Invitrogen) 1–2 units. Control reactions were done with the same procedure but only with *Platinum*^*®*^*Taq*. Amplification conditions: cDNA was formed in a 50 °C 30 min cycle. Then, an initial 94 °C 2 min denaturation cycle was followed by 25–35 30-s cycles at 95 °C. A final extension cycle of 10 min at 72 °C reaction was allowed.

### Construction of the complemented strains

In order to complement the *ncs*, *ncyP and tal*-deleted strains, each of those genes was obtained by PCR amplification, sequenced to confirm their integrity and subcloned downstream of the anhydrotetracycline-inducible *tcp*-830 promoter in the EcoRV site of the conjugative-integrative vector pAV11 [23]. The constructions were introduced in *E.coli* ET12567/pUZ8002, and then were transferred to the adequate *Streptomyces* strain by conjugation. Hygromycin resistant exconjugants were tested by PCR to confirm the presence of the complementing genes.

## Additional files

10.1186/s12934-015-0373-7 A) Conserved sequences around the key amino acids located in chalcone synthases active center. The numbers given in the sequence correspond to *S. clavuligerus* Ncs, where the key amino acids are Cys^138^, Phe^188^, His^270^ and Asn^303^. B) Phylogenetic three of plant and *Streptomyces* chalcone synthases. The origin of the sequences used is indicated at the right side. Numbers at the far right side correspond to the genetic distance index.
10.1186/s12934-015-0373-7 Oligonucleotides used in this work.
