# Supplementary material for: Molecular genetics of naringenin biosynthesis, a typical plant secondary metabolite produced by Streptomyces clavuligerus
Source: Microb Cell Fact. 2015 Nov 9;14:178. doi: 10.1186/s12934-015-0373-7 (PMC4640377; doi:10.1186/s12934-015-0373-7)
Supplement: Supplementary file 1 — 10.1186/s12934-015-0373-7 A) Conserved sequences around the key amino acids located in chalcone synthases active center. The numbers given in the sequence correspond to S. clavuligerus Ncs, where the key amino acids are Cys138, Phe188, His270 and Asn303. B) Phylogenetic three of plant and Streptomyces chalcone synthases. The origin of the sequences used is indicated at the right side. Numbers at the far right side correspond to the genetic distance index. [file 12934_2015_373_MOESM1_ESM.pdf]

A

	132 to 146	181 to 197	266 to 278	295 to 307
S. clavuligerus 5492	PIAQLGCAAGGAAVN	SLLSNGLFGDGIAAAVV	FYIIHAGGPRILD	RATLTEYGNIASA
S. coelicolor 1206	PIAQLGCAAGGAAIN	SLLCNGLFGDGIAAAVV	FYIVHAGGPRILD	RATLTEYGNIASA
S. avermitilis 7131	PIAQLGCAAGGSAIN	SLLSNGLFGDGIAAAAR	FYIIHAGGPRILD	RATLTEYGNIASA
S. ven 5367	PIAQLGCAAGGAAIN	SLLSNGLFGDAVAAAVV	FYIIHAGGPRILD	RATLTEYGNIASA
S. griseus 6620	PIAQLGCAAGGAAIN	SLLSNGLFGDALSAAVV	FFIVHAGGPRILD	RATLTERGNIASS
	* ** ***	** **** *	* * *** ***	* * * ** *
Glycine	MMYQQGCFAGGTVLR	SLVGQALFGDGAAAVIV	FWIAHPGGPAILD	RHVLSEYGNMSSA
Medicago	MMYQQGCFAGGTVLR	SLVGQALFGDGAAALIV	FWIAHPGGPAILD	REVLSEYGNMSSA
Citrus	MMYQQGCFAGGTVLR	SLVGQALFGDGAAAVIV	FWIAHPGGPAILD	RQVLSEYGNMPSA
Arabidopsis	MMYQQGCFAGGTVLR	SLVGQALFGDGAAALIV	FWIAHPGGPAILD	RHVLSEYGNMSSA
Zea Mays	MMYQQGCFAGGTVLR	SLVGQALFGDGAAAVVV	FWVAHPGGPAILD	RHVLSEYGNMSSA
	138	188	270	303

B

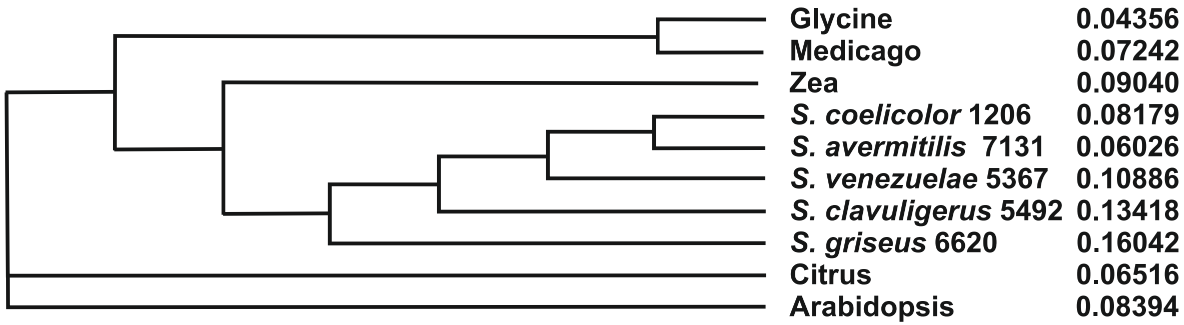

Supplementary Material

Figure S1. A) Conserved sequences around the key amino acids located in chalcone synthases active center. The numbers given in the sequence correspond to *S. clavuligerus* Ncs, where the key amino acids are Cys[138], Phe[188], His[270] and Asn[303].

B) Phylogenetic three of plant and *Streptomyces* chalcone synthases. The origin of the sequences used is indicated at the right side. Numbers at the far right side correspond to the genetic distance index.
