# Supplementary material for: Molecular genetics of naringenin biosynthesis, a typical plant secondary metabolite produced by Streptomyces clavuligerus
Source: Microb Cell Fact. 2015 Nov 9;14:178. doi: 10.1186/s12934-015-0373-7 (PMC4640377; doi:10.1186/s12934-015-0373-7)
Supplement: Supplementary file 2 — 10.1186/s12934-015-0373-7 Oligonucleotides used in this work. [file 12934_2015_373_MOESM2_ESM.doc]

Supplemental Information. Table S1. **Oligonucleotides used in this work**

| **Oligonucleotide** | **Sequence 5' to 3'** | **Description** |  |
| --- | --- | --- | --- |
| ***ncs*_RD_F** | GTCAGAGGGGCCATCCTGCGCTAGAAGAAGACTCCCATGATTCCGGGGATCCGTCGACC | Forward for *ncs* delection |  |
| ***ncs*_RD_R** | TCGACAACGTCTTCGGCGCCTACTGACCGACCGGGCTCATGTAGGCTGGAGCTGCTTC | Reverse for *ncs* delection |  |
| ***ncyP*_RD_F** | GCGCCCCGGGGCCGCCCCGTTGCGCAGCACGGATGAATGATTCCGGGGATCCGTCGACC | Forward for *ncyP* delection |  |
| ***ncyP*_RD_R** | GCGGGCACGGCGGGGCCGCCGGGCCCGCCCGGCCGTCACTGTAGGCTGGAGCTGCTTC | Reverse for *ncyP* delection |  |
| ***ncs*_F** | TCTCCTATCTGACCGCCAAC | Forward for *ncs* PCR |  |
| ***ncs*_R** | TCAACGACTGGGACATCAAC | Reverse for *ncs* PCR |  |
| ***ncyP*_F** | CGTTGCGCAGCACGGATGAATGAGG | Forward for *ncyP* PCR |  |
| ***ncyP*_R** | AGCGCACCGCGTAGGAGATCCACTC | Reverse for *ncyP* PCR |  |
| ***ncs*_CP_F** | CATGCCATGGCAGTACTGTGCAAAC | Forward for *ncs* complementation |  |
| ***ncs*_CP_R** | CCCAAGCTTCTACGGCTCCATCGACAAC | Reverse for *ncs* complementation |  |
| ***ncyP*_CP_F** | ATGAGGGGCGGCCATCCG | Forward for *ncyP* complementation |  |
| ***ncyP*_CP_R** | TCACCAGGTGACGGGCAG | Reverse for *ncyP* complementation |  |
| **RT_*ncs*_F** | GAGAGACCCGTCATGTCCAC | Forward for *ncs* RT-PCR |  |
| **RT_*ncs*_R** | AGGAGTCCTCCGCCATCC | Reverse for *ncs* RT-PCR |  |
| **RT_*ncyP*_F** | AACGAGGAGACCCTCGAACT | Forward for *ncyP* RT-PCR |  |
| **RT_*ncyP*_R** | CTGGTAGCACAGCGAACAGA | Reverse for *ncyP* RT-PCR |  |
| **RT_91_92_F** | GCTCGCACAGGCCGAGATGGAGACCGCCTTCAGGACGCT | Forward for coexpression RT-PCR |  |
| **RT_91_92_R** | TGCAGTCTCCGAGCCAGTTCGAGGG | Reverse for coexpression RT-PCR |  |
| ***tal*_RD_F** | CTAGCCTTGGCCGTCTCACGTCCGAGGTGATGCGATATGATTCCGGGGATCCGTCGACC | Forward for *tal* delection |  |
| ***tal*_RD_R** | TTCCGCTGATGAACGCGACCTTGCCTTCGAGGCGACCGGTGTAGGCTGGAGCTGCTTC | Reverse for *tal* delection |  |
| ***tal*_F** | GTGCGGAGTGAGGGCTTC | Forward for *tal* PCR |  |
| ***tal*_R** | AGCGTCTTGTCGGAGGTCT | Reverse for *tal* PCR |  |
| ***tal*_CP_D** | ATGTCTGCGATCGAAGTCGGC | Forward for *tal* complementation |  |
| ***tal*_CP_R** | TTGCCTTCGAGGCGACCG | Reverse for *tal* complementation |  |
| **Q _*tal*_D** | GGTGTGACGACGGGTTTC | Forward for *tal* quantitative RT-PCR |  |
| **Q _*tal*_R** | CGTTGAGGTGGTAGGTGATG | Reverse for *tal* quantitative RT-PCR |  |
| **Q _*ncyP*_D** | ACCGCGACCACAGCAC | Forward for *ncyP* quantitative RT-PCR |  |
| **Q _*ncyP*_R** | GCATAGTCGAAGGGGCAGTA | Reverse for *ncyP* quantitative RT-PCR |  |
| **Q _*ncs*_D** | TCCTGGCTCGTCAACACC | Forward for *ncs* quantitative RT-PCR |  |
| **Q _*ncs*_R** | GTAGGCGGTGCAGAAGTCAT | Reverse for *ncs* quantitative RT-PCR |  |
| **Q-hrdB_D** | cgcggcatgctcttcct | Forward for *hrdB* quantitative RT-PCR |  |
| **Q-hrdB_R** | aggtggcgtacgtggagaac | Reverse for *ncs* quantitative RT-PCR |  |
| **Q-hrdB_D** | cgcggcatgctcttcct | Forward for hrdB quantitative RT-PCR |  |
| **Q-hrdB_R** | aggtggcgtacgtggagaac | Reverse for ncs quantitative RT-PCR |  |
